# Neural mechanisms of interstimulus interval-dependent responses in the primary auditory cortex of awake cats

**DOI:** 10.1186/1471-2202-10-10

**Published:** 2009-02-10

**Authors:** Masashi Sakai, Sohei Chimoto, Ling Qin, Yu Sato

**Affiliations:** 1 Department of Physiology, Interdisciplinary Graduate School of Medicine and Engineering, University of Yamanashi, Yamanashi 409-3898, Japan

## Abstract

**Background:**

Primary auditory cortex (AI) neurons show qualitatively distinct response features to successive acoustic signals depending on the inter-stimulus intervals (ISI). Such ISI-dependent AI responses are believed to underlie, at least partially, categorical perception of click trains (elemental vs. fused quality) and stop consonant-vowel syllables (eg.,/da/-/ta/continuum).

**Methods:**

Single unit recordings were conducted on 116 AI neurons in awake cats. Rectangular clicks were presented either alone (single click paradigm) or in a train fashion with variable ISI (2–480 ms) (click-train paradigm). Response features of AI neurons were quantified as a function of ISI: one measure was related to the degree of stimulus locking (temporal modulation transfer function [tMTF]) and another measure was based on firing rate (rate modulation transfer function [rMTF]). An additional modeling study was performed to gain insight into neurophysiological bases of the observed responses.

**Results:**

In the click-train paradigm, the majority of the AI neurons ("synchronization type"; *n *= 72) showed stimulus-locking responses at long ISIs. The shorter cutoff ISI for stimulus-locking responses was on average ~30 ms and was level tolerant in accordance with the perceptual boundary of click trains and of consonant-vowel syllables. The shape of tMTF of those neurons was either band-pass or low-pass. The single click paradigm revealed, at maximum, four response periods in the following order: 1st excitation, 1st suppression, 2nd excitation then 2nd suppression. The 1st excitation and 1st suppression was found exclusively in the synchronization type, implying that the temporal interplay between excitation and suppression underlies stimulus-locking responses. Among these neurons, those showing the 2nd suppression had band-pass tMTF whereas those with low-pass tMTF never showed the 2nd suppression, implying that tMTF shape is mediated through the 2nd suppression. The recovery time course of excitability suggested the involvement of short-term plasticity. The observed phenomena were well captured by a single cell model which incorporated AMPA, GABA_A_, NMDA and GABA_B _receptors as well as short-term plasticity of thalamocortical synaptic connections.

**Conclusion:**

Overall, it was suggested that ISI-dependent responses of the majority of AI neurons are configured through the temporal interplay of excitation and suppression (inhibition) along with short-term plasticity.

## Background

The perceptual quality of successive acoustic signals considerably varies depending on the inter-stimulus intervals (ISI). For example, when click signals are repetitively presented at ISI ≥ ~30 ms, individual signals are clearly heard as discrete events [1]; at ISI ≤ ~30 ms, those are perceptually fused together [2,3]. This ISI boundary, denominated as "temporal-order threshold," has long been considered as an important indicator of temporal resolving capacity of the auditory system (reviewed by [4]). Another example of ISI-dependent perception is categorical perception of stop consonant-vowel syllables (CV syllables): if ISI between the consonant release and voicing onset (voice onset time [VOT]) is shorter than a critical value (VOT boundary), the consonant is perceived as "voiced"; if ISI exceeds this value, the consonant is perceived as "unvoiced" (eg.,/da/-/ta/continuum [5]). In many languages including English, the VOT boundary lies at 20–40 ms with some variance among place of articulation (reviewed by [6]). Monkeys [7], chinchillas [8] and birds [9] all place the VOT boundary at approximately the same value, indicating the categorical perception of CV syllables does not necessarily arise from a specific human speech mechanism but is based, at least partially, on general properties of the auditory system.

Case studies of patients with stroke lesions restricted to the bilateral primary auditory cortex (AI) reported that (1) their temporal-order threshold was elongated up to ~100 ms [10,11] and (2) they were severally impaired in the categorical perception of CV syllables [11,12]. These findings suggest that AI is critically involved in ISI-dependent differential perception regardless of whether the signals are phonetic or non-phonetic (reviewed by [13]).

The previous single unit study in un-anesthetized animals AI revealed that click trains produce qualitatively distinct response features depending on ISI: at ISI ≥ ~30 ms, stimulus-locking responses dominate; at ISI ≤ ~30 ms, responses occur only at the onset of the train [14]. Similar finding was obtained for AI responses to CV syllables: at ISI (VOT) ≥ ~30 ms, stimulus-locking responses take place to both the consonant and vowel; at ISI (VOT) ≤ ~30 ms, responses occur only to the consonant [15]. Since these neurophysiological ISI boundaries (~30 ms) match both the temporal-order threshold and VOT boundary (see above), it was suggested that the neural processes constraining AI stimulus-locking responses are also responsible for the perceptual boundaries of phonetic/non-phonetic acoustic signals [13].

The present study, by employing a single unit recording technique in un-anesthetized cats, thoroughly analyses how AI neurons respond to click trains of variable ISI. Then, by modeling the observed responses, we extract general principles governing various ISI-dependent behaviors of AI neurons especially stimulus-locking responses.

## Results

### Response Features in the Click-train Paradigm

The results are based on 116 AI neurons that showed statistically significant excitatory responses to the click stimuli (see Methods). We classified those neurons into 2 types depending on whether they had the capacity for stimulus-locking responses to click trains (synchronization type: n = 72) or not (non-synchronization type: n = 44).

**Figure 1:**
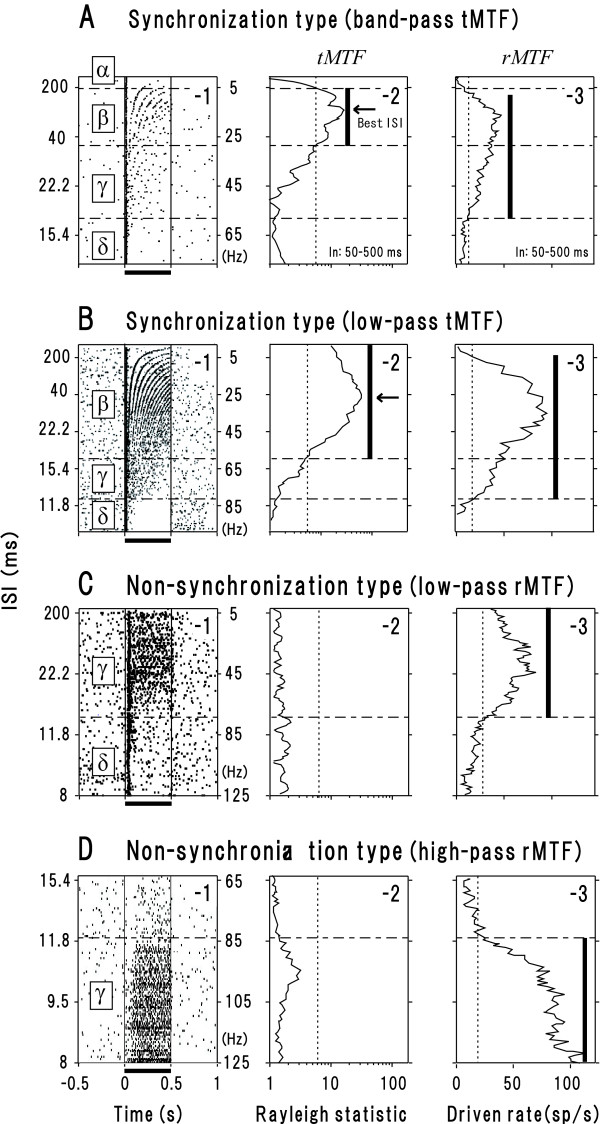
**Response profiles for 4 representative neurons (A, B: synchronization type; C, D: non-synchronization type) in the click-train paradigm**. (**1st column**) Raster display of spike occurrence in response to 0.5-s-long click trains (horizontal bar) at variable inter-stimulus intervals (ISI; ordinate, left) or repetition rate (ordinate, right). Regions *α*-*δ *represent qualitatively distinct response patterns as defined below. (**2nd column**) The "temporal modulation transfer function (tMTF)," defined as Rayleigh values as a function of ISI. The vertical bar denotes the ISI range of "region *β*" where statistically-significant degree of stimulus-locking responses took place (P < 0.05, Rayleigh statistics). The ISI that gives the maximal Rayleigh value is denoted as the "best ISI" (arrow). In majority of the synchronization neurons, the longer-ISI limit of region *β *(upper margin) was bordered with "region *α*" (**A-1**) where only the onset response was evident. (**3rd column**) The "rate modulation transfer function (rMTF)," defined as the mean driven rate (over 50–500 ms after the initiation of the train) as a function of ISI. The vertical bar denotes the ISI range where the mean driven rate exceeded the threshold for excitation (2*SD of spontaneous firing rate; dotted line). Among the ISI range, the one where no stimulus-locking responses took place was denominated as "region *γ*." In majority of the neurons examined, the shorter-ISI limit (lower margin) of region *γ *was bordered with "region *δ*" where only the onset response was evident. See text for details.

**Figure 2:**
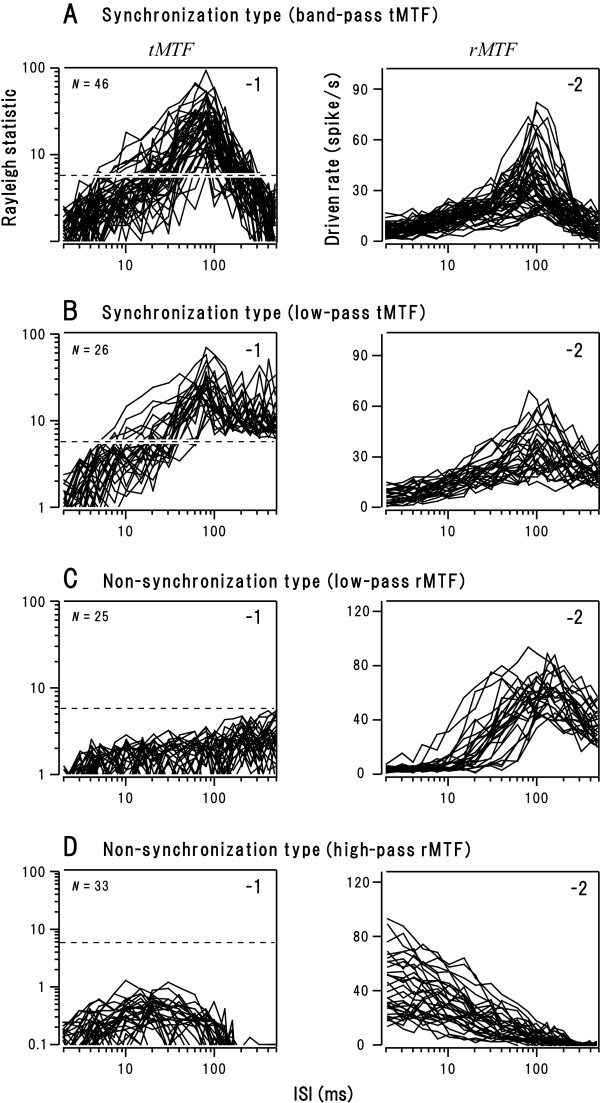
**The tMTF (left column) and rMTF (right column) for individual synchronization neurons with band-pass tMTF (A) or low-pass tMTF (B), and for non-synchronization neurons with low-pass rMTF (C) or high-pass rMTF (D)**. Dotted line: statistically significant level (P = 0.05) for the Rayleigh test. See Figure 1 and text for definition of the response variables.

As exemplified in Figure 1A, the majority of synchronization type neurons (n = 46) exhibited 4 qualitatively distinct response patterns (regions *α*-*δ*) depending on ISI. In region *α *(ISI ≥ 200 ms, A-1), only the onset response was evident. In region *β *(ISI: 38–200 ms) spikes clearly time-locked to individual clicks: temporal modulation transfer function (tMTF; see Methods) exceeded the statistically significant level (P < 0.05; A-2, dotted line). Hereafter, we call this response pattern "stimulus-locking responses." In region *γ *(ISI: 16–38 ms, A-1), spikes intermittently occurred without stimulus locking (A-2). The driven rate measured 50–500 ms after the onset of the train (rate modulation transfer function: rMTF; see Methods) exceeded the threshold for excitation (A-3, dotted line). In region *δ *(ISI ≤ 16 ms, A-1), the onset response was followed by an unresponsive period. Since region *β *of this subset was bordered with regions *α *and *γ*, we regarded tMTF shape as "band-pass" (A-2; summarized in Fig. 2A–). As exemplified in Figure 1B, the remaining 26 synchronization neurons exhibited 3 response regions. Since region *β *of this subset was bordered with only region *γ*(B-1), we regarded tMTF shape as "low-pass" (B-2; summarized in Fig. 2B–). Regardless of tMTF shape, the border between regions *β *and *γ *(hereafter, *β*-*γ *border), in other words, the shorter cutoff ISI for stimulus-locking responses, lay at on average ~30 ms (Table 1). This value is in line with the previous single unit studies in un-anesthetized animals AI [14].

**Table 1:** Response variables (mean ± S.D.) in the click-train paradigm

Neuron type	*N*	*α*-*β *border	Best ISI (region *β*)	*β*-*γ *border	*γ*-*δ *border
Synchronization	46	174 ± 112 ms	71.4 ± 19.6 ms	30.6 ± 25.9 ms	12.1 ± 13.5 ms
(band-pass tMTF)		(5.7 ± 8.9 Hz)	(14 ± 51 Hz)	(33 ± 38 Hz)	(83 ± 74 Hz)

Synchronization	26	__	80.6 ± 23.8 ms	33.1 ± 28.7 ms	11.4 ± 16.9 ms
(low-pass tMTF)		(13 ± 42 Hz)	(30 ± 34 Hz)	(87 ± 59 Hz)	

Non-synchronization	25	__	__	__	14.9 ± 11.7 ms
(low-pass rMTF)					(67 ± 85 Hz)

Non-synchronization	19	__	__	__	__
(high-pass rMTF)					

See Figure 1, and Methods for neuron classification and definition of the response variables. Numbers within parentheses represent the reverse of ISI.

The non-synchronization neurons comprised 2 subsets. One subset (n = 25), as exemplified in Figure 1C, exhibited 2 response regions (regions *γ *and *δ*) with low-pass rMTF (C-3; summarized in Fig. 2C–2). Another subset (n = 19), as exemplified in Figure 1D, exhibited only region *γ *with high-pass rMTF (Fig. 2D; summarized in Fig. 2D–). These subsets, especially the latter, have been scarcely uncounted under anesthetized conditions [28,49,50]. They were, however, excluded from the following analysis since the main interest here is to extract general principles governing stimulus-locking responses (see Background).

### Effects of the Stimulus Level

To examine effects of the stimulus level, we adhered to the click-train paradigm at various stimulus levels (in pe-SPL; see Methods). We examined 34 synchronization neurons, firing activities of which could be isolated long enough for the detailed analysis. Among them, 29 neurons exhibited responses at 20 dB below the best SPL while 20 neurons did at 40 dB below the best SPL.

**Figure 3:**
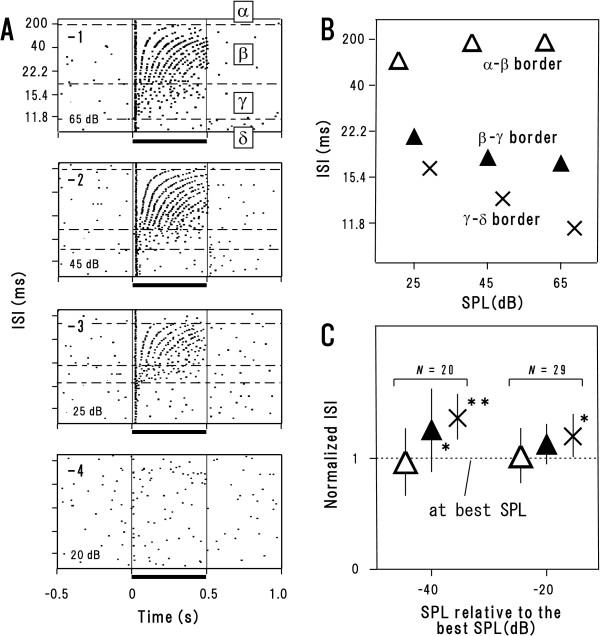
**Effects of the stimulus level on the synchronization neurons**. Responses of a representative neuron (**A**, **B**) and population data (mean ± SD) (**C**). **A-1 **to **-4**: Raster dot of spike occurrence at the best SPL (**-1**; in pe-SPL), 20 dB below (**-2**), 40 dB below (**-3**) and 45 dB below it (**-4**) (in the same format as Fig. 1A-1). **B: **The boundaries between the regions found in **A-1 **to **-3 **are plotted at each SPL. **C: **The population data for the same response variables as in **B**. The values were normalized to those measured at the best SPL (dotted line). *P < 0.05, **P < 0.01 (Fisher's PLSD test following one-way repeated ANOVA).

Figure 3A demonstrates responses of a representative neuron (best SPL: 65 dB). Regions *α*-*δ *were clearly identified at any stimulus level as long as statistically significant responses were elicited (Fig. 3A–1 to 3A–3; in the same format as Fig. 1A–1). However, the stimulus level, more or less, influenced the ISI values that divide the response regions. First, at 20 dB below the best SPL, the *α*-*β *border (Fig. 3B, open triangle, middle) and *β*-*γ *border (filled triangle, middle) were roughly the same as those measured at the best SPL (corresponding symbols, right); whereas the *γ*-*δ *border got slightly longer (cross, middle). Second, at 40 dB below the best SPL, the *α*-*β *border (open triangle, left) got slightly shorter whereas the *β*-*γ *border (filled triangle, left) as well as *γ*-*δ *border (cross, left) got longer. These observations are confirmed by the population data (Fig. 3C; the values were normalized to those measured at the best SPL).

### Accumulation Effects: Region *β*

It has been widely reported that repetitive stimulation exerts accumulation effects on auditory neurons especially those in the auditory cortex (reviewed by [16,17]). We addressed whether and how such effects influenced the observed responses. In the present and the following section, we paid special attention to regions *β *and *δ*, where the causative relationship between a given stimulus and spikes can be clearly identified.

**Figure 4:**
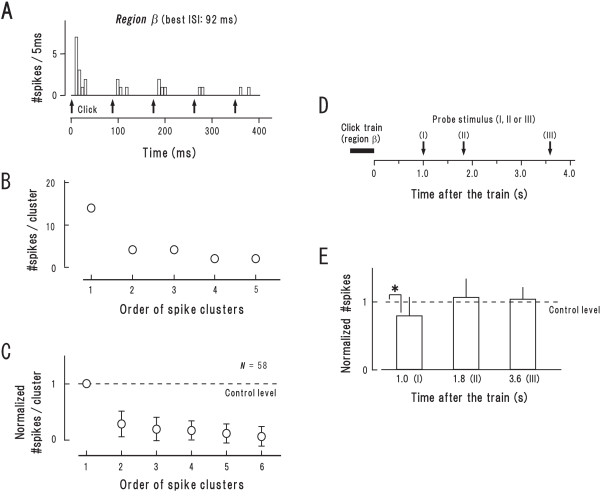
**Accumulation effect of responses in region *β *(at the best ISI) (A-C) and its durability (D, E)**. **A, B: **Peri-stimulus time histogram (bin width = 5 ms) (**A**) and sequential plot of the number of spikes (# spikes) involved in each discharge cluster (every discharge cluster was elicited by single clicks) (**B**). **C: **Population data (mean ± SD) in the same format as **B **except that #spikes was normalized to that involved in the 1st discharge cluster (= control level; same as followings). **D: **The procedure for measuring the responsivity after the trains that were delivered at the best ISI: a single click (= probe stimulus; arrows) was presented at either 1.0 s (I), 1.8 s (II) or 3.6 s (III) after the termination of the train. **E: **Population data of the normalized #spikes measured according to **D**. *P < 0.05 (paired t-test). Note (1) responsivity gradually declined with the progression of the train (**C**), and (2) the response degeneration lasted for 1.0-1.8 s after the termination of the train (**E**).

Figure 4A displays the number of evoked spikes (#spikes; bin width = 5 ms) of a representative synchronization neuron (identical neuron as in Fig. 1A) at the best ISI (92 ms; for simplicity, responses at this relative ISI value is uniformly adopted in the following analysis of region *β*). #Spikes in each discharge cluster progressively decreased (Fig. 4B) indicating that the impact of successive clicks cumulatively reduced the responsivity to the following signals. This finding is confirmed by the population data (Fig. 4C) where #spikes elicited by each click was normalized to that elicited by the 1st click (hereafter, control level).

To address the durability of this response degeneration, we presented a single click (= probe stimulus) at 1.0, 1.8 or 3.6 s after the termination of the click trains that were delivered at best ISI (Fig. 4D). Figure 4E illustrates the normalized #spikes (see Methods) at each time point (Roman numerals correspond to the probe stimuli depicted in Fig. 4D). The value was still smaller than unity (broken line) at 1.0 s but became equivalent to unity at 1.8 and 3.6 s (P < 0.05; Fisher's PLSD test following ANOVA). This indicates that the response degeneration lasted 1.0–1.8 s after the termination of the trains.

The above features, both during and after the click trains, correspond to the phenomenon, so-called "frequency-dependent depression" [18].

### Accumulation Effects: Region *δ*

At relatively long ISIs in region *δ*, spikes occasionally occurred after the onset responses (Fig. 1A–1). Such activities potentially hinder temporal precision in measuring the duration of the onset responses. Those activities were sufficiently suppressed at ISIs at or shorter than 0.9 multiples of the *γ*-*δ *border in all synchronization type neurons examined. This relative ISI value is uniformly adopted in the following analysis of region *δ*.

**Figure 5:**
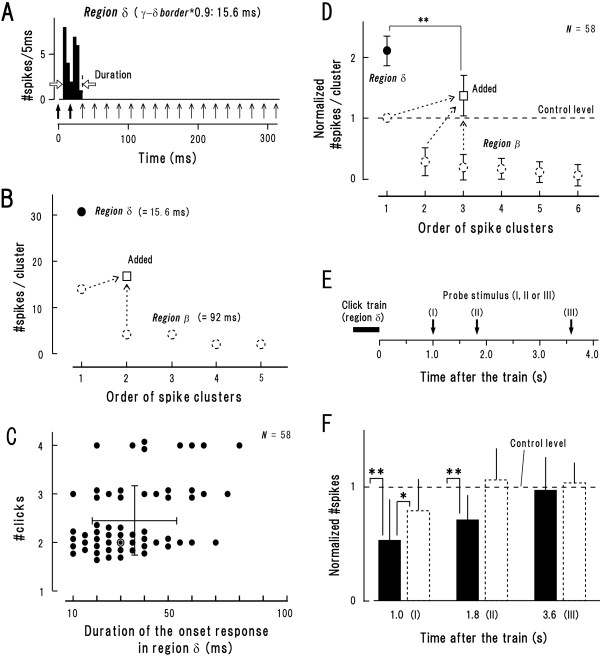
**Accumulation effect of responses in region *δ *(at 0.9 multiples of the *γ*-*δ *border) (A-D) and its durability (E, F)**. The format in **A**, **B**, **D**, **E**, **F **is the same as in Figure 4**A**-**E**, respectively. **A: **Peri-stimulus time histogram of the same neuron as in Figure 4**A**. The initial two clicks (arrows, bold) presumably contributed to the onset response (see text). **B: **#Spikes involved in the onset response (filled circle). For comparison, the data in Figure 4B was appended (in region *β*; broken circles) with the square representing #spikes elicited by the initial two clicks. **C: **Population data (mean ± SD) of joint distribution for the duration of the onset response (abscissa) and the number of clicks involved in it (#clicks, ordinate; mean = 2.4). The double circle represents the data in **A**. **D: **Population data of the normalized #spikes in region *δ *(filled circle). For comparison, the data in Figure 4C was appended (in region *β*; broken circles) with the square representing #spikes elicited by the initial three (> 2.4) clicks. **E: **Procedures for measuring the responsivity after the train that elicited region *δ *responses. **F: **Population data of the normalized #spikes measured according to **E **(filled bar). For comparison, the data in Figure 4**E **was appended (after region *β*; broken bar). *P < 0.05, **P < 0.01 (paired t-test).

Figure 5A displays #spikes (identical neuron as in Fig. 1A) at 15.6-ms ISI (region *δ*) which corresponds to 0.9 multiples of the *γ*-*δ *border. The onset response was 30 ms in duration (arrows, horizontal) into which 2 clicks fell (bold arrows, vertical) indicating that discharge clusters elicited by the 1st and 2nd click merged together. The onset response included 31 spikes (Fig. 5B, closed circle). This number was much larger than the total amount of #spikes which were elicited by the 1st and 2nd click in the trains delivered at the best ISI (square) (broken circles represent the same data as in Fig. 4B). The population data provides the similar findings: (1) the onset response in region *δ *was on average 36.8 ± 18.1 ms in duration (Fig. 5C, horizontal line with patch), into which 2.4 ± 0.7 clicks fell (vertical line with hatch); (2) the normalized #spikes involved in the onset response was on average 2.1 ± 0.3 (Fig. 5D, closed circle); (3) this value was significantly larger than the total amount of the normalized #spikes which were elicited by the initial 3 clicks (> 2.4 [= the mean number of clicks that contributed to the onset response in region *δ*; see above]) presented at the best ISI (square; broken circles denote the same data as in Fig. 4C) (P < 0.01, paired t-test). These findings suggest that when several consecutive clicks fall within a few tenths of millisecond, there is a synergy of impact. This phenomenon seemingly corresponds to "paired-pulse facilitation" [19].

The onset response in region *δ *was typically followed by an unresponsive period (Fig. 5A). To estimate the recovery time course from this response degeneration, we presented a probe stimulus at 1.0, 1.8 or 3.6 s after the termination of the train (Fig. 5E). The normalized #spikes at each time point was illustrated as filled bars in Figure 5F (same format as Fig. 4E). For comparison, the data in Figure 4E (broken bars) was appended, which depicts the recovery time course after region *β*. The major findings are: (1) at 1.0 s, the value was smaller after region *δ *than after region *β *(P < 0.05, t-test); (2) at 1.8 s, the value after region *δ *was still below unity (P < 0.05) while the value after region *β *got equivalent to unity. Together, it appears that response degeneration is more profound and longer lasting after region *δ *than after region *β*. This fits well to the principle of "frequency-dependent depression": response degeneration grows larger and longer at higher stimulation rates [18]. It is, thus, plausible that the unresponsive period in region *δ *was caused by such intense "frequency-dependent depression" as to abolish firing activities for a while.

### Involvement of Post-activation Suppression in Stimulus-locking Responses

The neural processes of stimulus-locking responses (e.g., Fig. 4A) can be glimpsed if we pay special attention to the vector strength, the origin of the tMTF (see Methods). The vector strength measures the degree of temporal confinement of spikes against stimuli. It reaches a maximum (= 1.0) when spikes occur in exactly the same period with reference to the individual stimuli, and spikes (regardless of whether evoked or spontaneous) are completely absent in the remaining period. On the other hand, it reaches a minimum (= 0.0) when spikes occur entirely independently of the stimuli. It is, thus, quite conceivable that the capacity for stimulus-locking responses arise from neural processes that temporally confine spikes. To examine this, we conducted the single-click paradigm in which the dynamics of neural activity after single click presentation was qualified with referring to the spontaneous firing rate (excitation, suppression or spontaneous-level activities; see Methods). This analysis was performed on 35 neurons (non-synchronization type, n = 8; synchronization type, n = 27) that showed an appreciable amount of spontaneous firing rate for detecting suppression.

**Figure 6:**
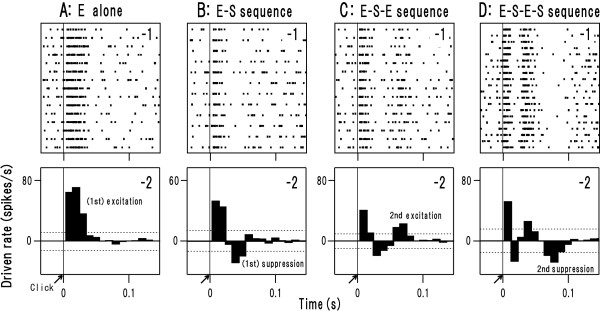
**Dynamics of firing responses of 4 representative neurons (A: non-synchronization type; B-D: synchronization type) in the single-click paradigm**. A single click (arrow) was presented, and the spike occurrence was examined with raster dot plots (**-1**) and post-latency time histogram (bin width = 10 ms; **-2**). The threshold for excitation and suppression was set at plus/minus 2*SD of the mean of spontaneous firing rate (dotted line), respectively. The qualitatively distinct responses were aligned in the following sequence: only excitation (**A**), excitation (E) followed by suppression (S) (hereafter, "E-S sequence"; **B**), "E-S-E sequence" (**C**) and "E-S-E-S sequence" (**D**).

Figures 6A–D show responses of 4 representative neurons (1: raster plots of spike occurrence; 2: post-latency time histograms, see Methods). As exemplified in Figure 6A, all non-synchronization neurons examined (low-pass rMTF, *n *= 4; high-pass rMTF, *n *= 4) showed only single excitatory period (1st excitation). In contrast, as exemplified in Figure 6B–D, all synchronization neurons showed a "post-activation suppression": the 1st excitation was followed by the suppression(s), suggesting the critical role of post-activation suppression in stimulus-locking responses.

We sorted the synchronization neurons into 3 subsets based on the sequence of excitation and suppression (Table 2, rightmost column). The smallest subset (*n *= 5) showed "E-S sequence" (eg., Fig. 6B–2): only the 1st excitation and 1st suppression were evident. The largest subset (*n *= 13) showed "E-S-E sequence" (eg., Fig. 6C–2): the 1st suppression was followed by a rebound excitation (2nd excitation). This sequence has been often observed in AI [20,21] and somatosensory cortex [22]. The remainder (n = 9) showed "E-S-E-S sequence" (eg., Fig. 6D–2): the 2nd excitation was followed by another suppressory period (2nd suppression). This subset includes 3 neurons in which the 1st and 2nd suppression were separated by spontaneous-level activities instead of the 2nd excitation. Among the synchronization neurons examined, the 2nd suppression was present in 9 out of 14 neurons with band-pass tMTF, whereas it was absent in all the neurons with low-pass tMTF (Table 2). Fisher's exact test revealed a significant effect of the 2nd suppression on the tMTF shape (P < 0.001). It is, thus, plausible that the interplay of the 2nd excitation and 2nd suppression constrains tMTF shape (band-pass vs. low-pass).

**Table 2:** Sequence of response periods observed in the single-click paradigm

Neuron type	*N*	1st E	1st S	2nd E	2nd S	Sequence
Synchronization	9/14	+	+	+/-	+	E-S-E-S
(band-pass tMTF)	5/14	+	+	+	-	E-S-E

Synchronization	8/13	+	+	+	-	E-S-E
(low-pass tMTF)	5/13	+	+	-	-	E-S

Non-synchronization	8	+	-	-	-	E

E: excitation; S: suppression.

### Modeling of AI Temporal Behavior

**Figure 7:**
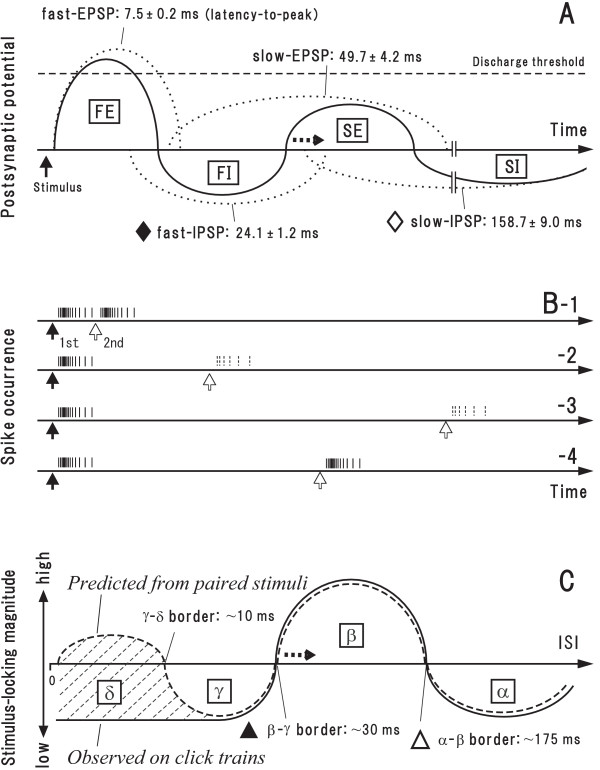
**A: Scheme for a sequence of EPSPs and IPSPs (broken curves) in the AI neuron elicited by a single afferent stimulation (based on **[27]**)**. Temporal interaction between the PSPs may form four phases (solid curves) in the following order: fast excitatory (FE), fast inhibitory (FI), slow excitatory (SI), and slow inhibitory (SI) periods. Numerals represent latency-to-peak (mean ± SD). Identical time scale is applied to **A**-**C**. **B: **Predicted firing responses (vertical lines) elicited by paired stimuli of various ISI. When the 2nd stimulus is given during period FE (ref., **A**; same as followings) (**-1**), it would readily elicit firing responses. However, the discharge clusters elicited by the 1st and 2nd stimulus may merge together so that stimulus-locking responses, if any, would be weak. When the 2nd stimulus is given during period FI (**-2**) or SI (**-3**), it would hardly elicit firing responses and, consequently, stimulus-locking responses. When the 2nd stimulus is given during period FE (**-4**), it would readily elicit firing responses. The discharge clusters elicited by the 1st and 2nd stimulus may be clearly separated each other by period FI leading to intense stimulus-locking responses. **C: **The tMTF that derived from the prediction in **B **(broken curve) and the tMTF that was schematically drawn from the data of the synchronization type with band-pass tMTF (solid curve; based on Table 1). Regions *α*-*δ *correspond to those in Figure 1A-1. Enhancement of GABA_A_-ergic inhibition may shift the FI-SE transition point afterward (A, horizontal arrow) thus prolonging *β*-*γ *border (C, horizontal arrow).

de Ribaupierre and colleagues [23] reported in AI single unit study that stimulus-locking responses were greatly related to the temporal interplay of depolarization and hyperpolization. Compelling evidence indicates that depolarization and hyperpolization of AI neurons are chiefly based on excitatory post-synaptic potentials (EPSPs) and inhibitory post-synaptic potentials (IPSPs), respectively [24-27]. Notably, Cox and colleagues [27] demonstrated in AI slice preparations that electrical stimulation to thalamocortical afferent fibers (Fig. 7A, arrow) elicits at maximum 4 PSP components at the soma of a subset of AI neurons in the following order: a fast-EPSP, fast-IPSP, slow-EPSP and slow-IPSP (broken curves). This scheme leads to the following prediction how the AI neurons generate firing responses to paired stimuli of variable ISI. First, when the 2nd stimulus (Fig. 7B–1, open arrow; in the same time scale as in Fig. 7A) is given during the fast-EPSP, it may readily elicit firing responses. However, the gap between the discharge clusters elicited by the 1st and 2nd stimulus is seemingly ambiguous.

Consequently, stimulus-locking responses, if any, would be weak (Fig. 7C, broken curve, region *δ*). Second, when the 2nd stimulus is given during the fast-IPSP (Fig. 7B–2) or slow-IPSP (Fig. 7B-3), it may hardly elicit firing responses. Stimulus-locking responses would be negligibly small (Fig. 7C, broken curve, region *γ *and region *α*). Third, when the 2nd stimulus is given during the slow-EPSP (Fig. 7B–4), it may readily elicit firing responses. Those discharge clusters are expectedly separated by the fast-IPSP (ref., Fig. 7A). As a consequence, clear stimulus-locking responses would take place (Fig. 7C, broken curve, region *β*). Interestingly, the "predicted tMTF for paired stimuli"(broken curve) resembles the "observed tMTF on click trains" that was schematically drawn based on the data of the synchronization type with band-pass tMTF (solid curve; based on Table 1) except for the short ISI potion (shaded zone). This inconsistency may arise from the fact that if the stimuli are repetitively delivered at short ISI, impact of initial several stimuli leads to such intense "frequency-dependent depression" as to abolish firing responses to the following stimuli (Fig. 5A). Collectively it is suggested that (1) as a principle, the temporal interplay of the PSP components underlies AI stimulus-locking responses and (2) at short ISI, intense frequency-dependent depression abolishes stimulus-locking responses.

**Figure 8:**
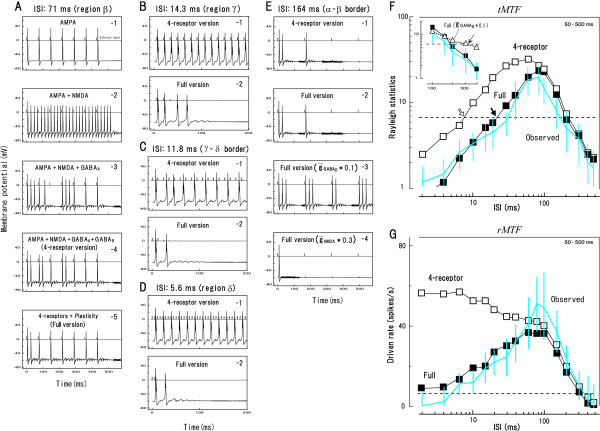
**A single-cell dynamic model replicates the response kinetics of the synchronization neurons**. The simulation was made on various combinations of the receptors (AMPA, GABA_A_, NMDA and GABA_B _receptors) with/without frequency-dependent depression (FDD) and paired-pulse facilitation (PPF) (depicted in each panel). Measures were generated by averaging responses over 20 simulations for each condition. **A**-**E: **Membrane potentials obtained at ISI of 71 ms (at the best ISI, region *β*; **A**), 14.3 ms (region *γ*; **B**), 11.8 ms (*γ*-*δ *border; **C**), 5.6 ms (region *δ*; **D**) and 164 ms (*α*-*β *border; **E**). **F**, **G: **The tMTF (**F**) and rMTF (**G**) obtained under various conditions. Measures were filtered by a weighted average with its 5 neighbors in the ratio of 1:2:3:2:1. Gray curve: the mean (with SD) of physiological data for the synchronization type with band-pass tMTF (based on 2A-1 for Fig. 8**F**, and 2A-2 for Fig. 8**G**). In **F**, the broken line denotes statistically significant level (P ≤ 0.05; Rayleigh Z-test) and the arrows indicate *β*-*γ *border (open, filled) or *α*-*β *border (dotted) in various conditions. See Methods for details.

To numerically examine the above conjecture, we conducted a simulation study (see Methods for details). The model consists of a single AI neuron which receives external input via various combinations of AMPA, GABA_A_, NMDA and/or GABA_B _receptors, each of which was reported to chiefly mediate the fast-EPSP, fast-IPSP, slow-EPSP and slow-IPSP, respectively (Fig. 7A, dotted curves [27]). The amplitude of external input was modeled in two different ways: (1) taking fixed value regardless of ISI or (2) varying in an ISI dependent manner due to both frequency-dependent depression and paired-pulse facilitation. Each panel in Figure 8A shows a version of simulated membrane potentials responding at the best ISI (= 71 ms). If AMPA receptors alone were incorporated into the model (Fig. 7A-1), then each input signal elicited firing responses. However, robust onset responses, which were always observed in region *β *(Fig. 4A), was unclear. If NMDA receptors were added into the model (Fig. 8A–2), then firing responses occurred throughout the stimulus train; however, stimulus locking became unclear. If GABA_A _receptors were added to the model (Fig. 7A-3), then the neuron gained the capacity for stimulus locking. While this feature, more or less, resembled our physiological observation (Fig. 4A), the former differed from the latter in that: (1) the 3rd stimulus failed to elicit firing responses and (2) the discharge clusters elicited by the 4th and 5th stimulus merged together. These discrepancies were diminished if GABA_B _receptors were incorporated into the model (Fig. 8A–4; "4-receptor version").

Nonetheless, the 4-receptor version does not necessarily parallel our physiological observation. First, this model predicts the occurrence of stimulus locking at much shorter ISI (e.g., 14.3 ms ISI; Fig. 8B–1) compared to physiological observation. In other words, the predicted *β*-*γ *border (4.5 ms; Fig. 8F, open arrow) was much shorter than the observed one (~30 ms, Table 1). Second, this model predicts "skipping" of firing responses at short ISI: for example, the spikes were expected to occur every other stimulus at 11.8 ms ISI (Fig. 8C–1) and every three stimuli at 5.6 ms ISI (Fig. 8D–1). These features, however, have been scarcely encountered in our physiological recording (but [22] in somatosensory cortex of anesthetized rats). In fact, the shortening of ISI led to the systematic reduction of responsivity to the latter clicks in a given train (e.g., Fig. 4A) while responsivity to the initial several clicks being relatively well preserved, as reported by plenty of single unit studies using periodic signals [14,20,23,28,29,38]. These two discrepancies were diminished when we provided the external input with both frequency-dependent depression (FDD) and paired-pulse facilitation (PPF) ("full version"). Specifically, the capacity for stimulus locking at shorter ISI was considerably weakened (cf., Fig. 8B–2 to 8B–1, 8C–2 to 8C–1, and 8D–2 to 8D–1) with only the onset response being manifested at the *γ*-*δ *border (Fig. 8C–2) and in region *δ *(Fig. 8D–2). As a consequence, the *β*-*γ *border prolonged to 25 ms (Fig. 8F, filled arrow).

At longer ISI, in marked contrast to short ISI, the 4-receptor version and full version predicted similar response features. For instance, at 164 ms the initial two clicks elicited stimulus-locking responses while the following ones did not (Fig. 8E–1,-2), similarly to region *α *(Fig. 1A–1). This indicates that the frequency-dependent depression and paired-pulse facilitation are much less influential at longer ISI.

Next, we investigated the main constraints on responses at long ISI. First examined was the contribution of GABA_B_-receptor-mediated IPSP. When the conductance of the GABA_B _receptors ($\bar{\text{g}}$_GABAB_) was reduced by factor of 0.1 (Fig. 8E–3; conductance of the other receptors was kept constant, same as followings), responsivity to the initial several stimuli was enhanced (cf., Fig. 8E–2) thereby prolonging the *α*-*β *border (Fig. 8F, inset, dotted arrow). Contrariwise, when the conductance of the NMDA receptors ($\bar{\text{g}}$_NMDA_) was reduced, responsivity to the 2nd and latter stimuli decreased (Fig. 8E–4) enhancing low-cut effect. Manipulation of the conductance of the AMPA or GABA_A _receptors did not greatly influence responses at long ISI (data not shown). Taken together, it is suggested that the $\bar{\text{g}}$_GABAB _and $\bar{\text{g}}$_NMDA _act as main constraints on the temporal filtering at long ISI: if the former is reduced, the tMTF tends to be more low-pass whereas if the latter is reduced, the tMTF more band-pass.

## Discussion

By using click signals, we investigated neural mechanisms underlying ISI-dependent responses of the AI neurons which had the capacity for stimulus-locking responses (synchronization type; Fig. 1A and Fig. 1B). The *β*-*γ *border, i.e., the shorter cutoff ISI for stimulus-locking responses, lay at on average ~30 ms (Table 1) and was level tolerant over high SPLs (Fig. 3C). The time course of excitability during (Figs. 4C and 5D) and after (Fig. 5F) the click trains suggested the involvement of short-term plasticity of thalamocortical synaptic connections. Comparison between response features to the click trains and a single click (Table 2) led to the notion that the temporal interplay of excitation and suppression basically determines the capacity for stimulus-locking responses as well as tMTF shape. A single-cell dynamic model well replicated the physiological data (Figs. 8F and 8G) suggesting that ISI-dependent responses of the synchronization neurons are configured through the temporal interplay of the post-synaptic potentials (Fig. 7A) along with short-term plasticity of thalamocortical synaptic connections.

### Perceptual Relevance of the Observed AI Responses

Case studies about AI-impaired patients indicated that AI is responsible for the temporal-order threshold (see Background). The mean value of the *β*-*γ *border (~30 ms; Table 1), i.e., shorter cutoff ISI for stimulus-locking responses (e.g., Figs. 1A–1 and 1A–2), agree well with the temporal-order threshold [4]. The *β*-*γ *border was nearly invariant at the best SPL and 20 dB below it (Fig. 3C, dotted line and filled triangle in the right, respectively) in accordance with the level tolerance of the temporal-order threshold over high SPL [30]. These findings strongly support the notion that the *β*-*γ *border serves as a neural correlate of the temporal-order threshold. Accordingly, it could be postulated that click trains are clearly heard as a series of "discrete" events as long as stimulus-locking responses dominate in AI. By taking count of the view that the temporal-order threshold and perceptual boundary of CV syllables at least partially share common neural processes (see Background), it is possible that the *β*-*γ *border also serves as the basis for the perceptual boundary of CV syllables.

The psychological studies have revealed that a total body of click trains of ISI ≥ ~30 ms produces two kinds of sensation: at ISI of ~30–200 ms, it leads to "rhythm" percept; at ISI ≥ ~200 ms, rhythm percept fades away while the sensation of "fluctuation" remains [1]. Since the value of the *α*-*β *border (~175 ms; Table 1) as well as its level tolerance (Fig. 3C, open triangles) is consistent with the rhythm-fluctuation boundary (~200 ms), it is possible that region *α *dominantly represents "fluctuation" whereas region *β *does "rhythm" percept.

At ISI < ~30 ms where individual clicks are no longer clearly heard as discrete events, a total body of click trains leads to three kinds of sensation with partial overlap [31-33]. At ISI of ~3–30 ms, a buzz or rattle like sensation is produced, defined as "roughness"; at ISI of ~5–15 ms, tone quality of sensation dominates, whose perceived frequency is directly related to waveform periodicity (defined as "periodicity pitch"); at ISI < several ms, another mode of pitch sensation dominates, which depends on the fundamental frequency ("spectral pitch"). None of the ISI range of these sensations does not fit to that of region *γ *or region *δ *(Table 1) making it unlikely that our single unit data have direct relevance to these sensations. Steinschneider and colleagues [34] suggested that periodicity pitch may be represented in AI by oscillatory neuronal ensemble responses locking to temporal envelope, and spectral pitch by rate-place coding that is sensitive to both the fundamental frequency and other harmonics in the train. Further insight into neurophysiological bases of these sensations would be obtained by coordinated single/multiunit recordings and psychoacoustic experiments.

### Comparison to Previous AI Studies in Un-anesthetized Animals regarding to Cell-type Classification

An accumulating body of single unit studies in un-anesthetized animals has investigated AI responses to periodic acoustic signals such as click trains [14,23] and amplitude-modulated sounds [35-38]. Irrespective of methological differences (e.g., stimulus configuration, electrode properties and statistics), these studies appear to agree that AI neurons comprise largely two subsets: one subset responds predominantly at long ISI (≥ ~30 ms, occasionally extending to 10 ms or less) in a stimulus-locking manner (i.e., temporal code) while another subset does at short ISI (< ~10 ms) in a sustained manner (i.e., rate code).

The following three findings indicate that our synchronization neurons (eg., Fig. 1A) correspond to the subset that was reported to conduct temporal code. First, the shorter cutoff ISI for stimulus-locking responses in those studies (30–40 ms) was similar to the *β*-*γ *border of our synchronization neurons (~30 ms; Table 1). Second, at shorter ISI (10–30 ms), the studies reported that the neurons intermittently fired without stimulus locking. This feature resembles region *γ *responses of our synchronization neurons. Third, at much shorter ISI (≤ 10 ms), the studies reported that only the onset response was evident. This feature, as well as its cutoff ISI, is quite akin to region *δ *of our synchronization neurons.

Our non-synchronization neurons with high-pass rMTF showed non-stimulus-locking responses during the presence of the click trains (eg., Fig. 1D). Such responses occurred only at short ISI (< ~10 ms) with shorter ISI leading to larger driven rate (D-3). This feature closely resembles the responses of the subset that was reported to conduct rate code. On the other hand, our non-synchronization neurons with low-pass rMTF (eg., Fig. 1C) do not correspond to either subset mentioned above. They may belong to the "unclassified neurons" in Wang and colleagues' study [14], which were reported to respond in some manner to click signals without clearly defined stimulus-locking responses or non-stimulus-locking rate responses.

### Comparison to Previous AI Studies of Neural Mechanisms underlying Stimulus-locking Responses

de Ribaupierre and colleagues [23] revealed that AI stimulus-locking responses are related to the temporal interplay of depolarization and hyperpolization. While this interplay potentially results from non-synaptically mediated after-hyperpolarization [39], growing evidence indicates that this interplay is based mainly on the sequence of EPSPs and IPSPs [25,26]. In particular, Cox and colleagues [27] proved in rat AI slice preparations that EPSPs and IPSPs are mediated chiefly through AMPA/NMDA receptors and GABA_A_/GABA_B _receptors, respectively (Fig. 7A). To date, however, it has been unclear whether and how these PSP components are related to stimulus-locking responses of AI neurons.

On the other hand, there is physiological data suggesting that AI stimulus-locking responses are mediated through other neural processes than the interplay of PSP components. For example Wehr and Zador [26], by employing the whole-cell recording technique on ketamine-anesthetized rats AI, measured the excitatory and inhibitory synaptic conductances elicited by click pairs of variable ISI. They found that inhibitory conductances were too short-lived to account for suppression of spiking responses to the 2nd click which was delivered at ISIs ≥ several hundreds milliseconds. Eggermont [40], based on physiological data, proposed a model in which presynaptic facilitation and depression determine the low-pass characteristics of AI stimulus-locking responses.

Our single-cell dynamic model comprehensively integrates the above findings/suggestions in that the capacity for stimulus-locking responses (i.e., region *β*) is explained in terms of the temporal interplay of the PSP components along with short-term plasticity (Fig. 8A–5). This view is compatible with the pioneering work of Grothe [41] that proved the critical contribution of EPSPs and IPSPs to stimulus-locking responses of auditory brainstem neurons for encoding interaural time difference. Furthermore, our model can explain the neural processes that give rise to the other ISI-dependent AI responses such as in regions *γ*, *δ *and *α *(Figs. 8B–2, D–2, and 8E–2).

It has been proved, in a gap-in-noise detection paradigm where leading and trailing wideband noise had the same frequency content, that the minimum gap between the noises (i.e., ISI) for stimulus-locking responses was 30 ms for a 20-ms leading noise, 10 ms for a 50-ms leading noise, and reached an asymptote of 5 ms for a 200-ms leading noise [15,42]. This implies that temporal resolving capacity of individual AI neurons is not fixed, but varies dynamically as depending on the duration of the leading signal. Since our model was based on the physiological data obtained using only 1-ms long clicks, this is best suited for neural processes that are triggered at the stimulus onset, not later in the stimulus.

The present study revealed that recovery period of AI spiking activities was in the order of seconds (Fig. 5F). This range is consistent with the values reported in awake (> 1s [43]) and ketamine-anesthetized animals (> 500 ms [26]) but is much longer than those measured in barbiturate-anesthetized animals (20–200 ms [44-46]). A number of observations indicate that barbiturate reduces spontaneous and evoked spiking activities [28,47,48]. This leads to a conjecture that frequency-dependent depression, which results chiefly from temporal exhaustion of readily releasable neurotransmitter pool [18], is much less potent and less durable under barbiturate anesthesia than the other conditions. Under barbiturate anesthesia, weight of the influence on the recovery period may shift from frequency-dependent depression, which lasts for seconds [17,18], to IPSPs which extend for maximally several hundreds milliseconds.

### Relative Contribution to AI Stimulus-locking Responses: Intra-AI Processing vs. Sub-AI Processing

There is a marked resemblance between the predicted tMTF (Fig. 7C, broken curve) and observed tMTF (solid curve) except for the short ISI portion (shaded area; for reason, see above). Remarkably, these tMTF curves derived from rather different experimental conditions: the former was based on the data obtained by electrical stimulation to rat AI slice preparations (Fig. 7A); the latter, during acoustic stimulation in un-anesthetized cats. By taking account of a large difference in the shorter cutoff ISI for stimulus-locking responses (i.e., *β*-*γ *border; Fig. 7C, filled triangle) between un-anesthetized (~30 ms; present study and [14]) and anesthetized animals (≥ 50 ms [28,49,50]), it seems likely that the neural processes of AI stimulus-locking responses are much less susceptible to the mechanical infringement of slicing the brain and species difference than the pharmacological effects of anesthetics. Some anesthetics, such as pentobarbital, are known to potentate GABA_A_-ergic inhibition [48]. This may enhance the fast-IPSP and consequently diminish the slow-EPSP, especially in its early phase (ref., Fig. 7A). As a result, the transition point from the fast-IPSP to slow-EPSP will shift afterward (horizontal arrow), prolonging *β*-*γ *border (Fig. 7C, horizontal arrow). Slice preparations, on the other hand, do not suffer such artificial enhancement of GABA_A_-ergic inhibition but retain local circuitry. These considerations favor the idea that AI stimulus-locking responses are elaborated mainly through intra-AI processing rather than simple preservation of sub-AI processing. The above idea receives support from the previous studies that directly compared the best ISI within pairs of functionally connected medial geniculate body (MGB) neuron and AI neuron [37,51]. In those studies, spiking activities of individual MGB and AI neurons were simultaneously recorded and the functional connection was confirmed if their activities showed a single cross-correlogram peak within 1–5 ms lag time, the MGB neuron leading the AI neuron, under both spontaneous and stimulus-driven conditions. Importantly, no rank correlation was revealed for the best ISI; MGB neurons with longer (shorter) best ISI did not preferentially connect with AI neurons with longer (shorter) best ISI. This suggests that the generally observed prolongation in the best ISI from MGB to AI [52,53] cannot be simply attributed to a degradation of temporal resolution due to intrinsic membrane properties or synaptic delay but is rather due to more elaborated intra-AI processing.

## Conclusion

Our physiological observation suggest that *β*-*γ *border, the shorter cutoff ISI for stimulus-locking responses of AI neurons, serves as a neural correlate of the temporal-order threshold and VOT boundary of CV syllables. The present modeling study supports the idea that the observed ISI-dependent responses are largely mediated through temporal interplay between EPSPs and IPSPs at the thalamocortical synapse along with its short-term plasticity.

The parameter values in our model were not directly measured in the current recording study but were determined referring to other published data (see Methods). Under this proviso, the weight of AMPA, GABA_A_, NMDA and GABA_B _receptors was arranged to better predict the observed phenomena. In fact, relative weight of the receptors may vary across neurons, and other cellular and/or network mechanisms may also contribute to the observed phenomena. Further insight into this issue would be glimpsed by measuring the membrane potential of the AI neurons during acoustic stimulation or analyzing the effect of selective receptor antagonists on their stimulus-response features.

## Methods

Experiments were performed in a manner consistent with the Guidelines for Animal Experiments, University of Yamanashi, and the Guiding Principles for the Care and Use of Animals approved by the Council of the Physiological Society of Japan. Animal preparation, recording, and histology procedures were the same as in our previous report [54-57]. Briefly, the cats underwent surgery under pentobarbital sodium anesthesia. Aluminum cylinders and metal blocks were implanted for subsequent extracellular recording and restraining of the animal's head, respectively. At least two weeks were allowed for recovery before the recording. During the recording, the animal was placed in an electrically shielded and sound-attenuated room with its body wrapped in a cloth bag and its head restrained with a holding bar. The animals were kept awake throughout the recording period and were monitored with an online surveillance camera and electroencephalography. When drowsiness was suspected, the cat was awakened by gently tapping its body using a remote-controlled tapping tool or by briefly opening the door of the room. A glass microelectrode (tip diameter, 1.8–2.5 *μ*m; resistance, 2–3 MΩ; filled with 2 M NaCl) was inserted into AI. Tone bursts of variable frequencies and sound pressure levels (SPLs) were presented as search signals. Single unit activities were recorded and their occurrences were identified using a window discriminator. The spike-occurrence outputs were captured on a Pentium-based computer with a time resolution of 2 *μ*s as the digital input for data analysis. The animals sometimes voluntarily moved during recording sessions, creating artifacts in the recording. By carefully checking the online monitor screens of the animal and firing activities, these motion artifacts were marked in real time on the recording-computer while recordings were in progress. Data with artifacts were rejected. Daily recording sessions lasted < 6 hours, and the total duration of the experiment continued for 2–6 months per animal. At the termination of the experiment, some recording sites were marked with electrolytic lesions. The animals were then sacrificed with an overdose of pentobarbital sodium and perfused with 10% formalin. The brain was cut in transverse sections and stained with neutral red. The recording sites were reconstructed based on the electrolytic lesions and electrode tracks.

### Sound generation and delivery

The sound signals were generated using user-written programs in MATLAB (Mathworks, Natick, MA) on a Pentium-based computer. The signals were fed into a 12-bit digital-to-analogue converter (BNC2090; National Instruments, Austin, TX) at a sampling interval of 100 kHz and to an eight-pole Chebyshev filter (P-86; NF Electric Instruments, Yokohama, Japan) with a high cutoff frequency of 20 kHz. The output was attenuated and sent to a low-output-impedance power amplifier (PMA2000III; Denon Electronic GmbH, Ratingen, Germany), and then the sound signals were presented from a speaker (K1000; AKG Acoustics, Wien, Austria) placed 2 cm away from the auricle contralateral to the recording site. We equalized and calibrated the sound delivery system between 128 and 16,000 Hz in 8 Hz steps, and the output varied by +/-1.5 dB. One set of stimuli was presented at variable intervals ranging 4.0–5.5 s. We employed (1) click-train paradigm and (2) single-click paradigm (see below).

### Click-train paradigm

Once single unit activities were isolated, we conducted a click-train paradigm: rectangular clicks (1-ms duration) were delivered in a train fashion (0.5-s duration; Fig. 1A–1, horizontal line). Since the neurons examined in the present study are included in our previous study for periodicity coding [57], click repetition rate was systematically varied at 0.5 (or 1.0, 2.0 or 4.0) Hz steps over 2.5–100 (or 2.5–480) Hz. Typical repetition rates were 2.5, 3.0, 3.5, 4.0, 4.5, 5.0, ... 99.0, 99.5, 100 Hz (Fig. 1B–1, ordinate, right). Consequently, inter-stimulus intervals (ISI), which is the reciprocal of the repetition rate, varied 10.0, 10.1, 10.2, ... 200, 222, 250, 333, 400 ms (ordinate, left). The intensity of the clicks for 20 repetitions (peak equivalent sound pressure level [pe-SPL]) was set at 20–80 dB (10 dB steps; occasionally 5 dB steps). An identical set of signals was presented 5–20 times. The analytic time window was set at 50–500 ms after the onset of the click trains for eliminating nonspecific effect of the onset response that takes place regardless of ISI value (see below). This kind of compensation has been often adopted in the previous AI studies [14,28,58,59].

Two measures were used to characterize response features at each SPL. The first was related to the degree of stimulus locking. At the beginning, the stimulus-locking discharges were quantified with vector strength [60]. The vector strength is calculated by the following equation:

$$VS=\frac{\sqrt{{\left(\sum_{\text{i}}^{n}x_{\text{i}}\right)}^{2}+{\left(\sum_{\text{i}}^{n}y_{\text{i}}\right)}^{2}}}{n}$$

where x_i _= cos *θ*_i _and y_i _= sin *θ*_i_, *n*: total number of spikes, and each spike is treated as a unit vector with a given phase 0–2 π assigned to the ISI of interest. The vector strength ranges between 0.0–1.0. A value of 0.0 indicates that spike occurrence is entirely independent of the signal periodicity, whereas a value of 1.0 indicates that all spikes occur at exactly the same phase as the signal. The significance of the vector strength was assessed using the Rayleigh Z-test [61] at the 5% significance level (Fig. 1A–2, dotted line). At a given SPL, Z value was filtered by a weighted average with its 5 neighbors in the ratio of 1:2:3:2:1 and was plotted against ISI (hereafter, "temporal modulation transfer function [tMTF]"). By comparing the tMTF obtained at different SPLs, we defined the value of the SPL and ISI to produce the maximum Z value as the "best SPL" and "best ISI" (arrow), respectively. Note, the following data were obtained at the best SPL, unless otherwise specified. If the maximum Z value of a given neuron exceed the significance level, we evaluated stimulus-locking responses taking place (solid bar) and classified the neuron as "synchronization type"; if not, classified as "non-synchronization type."

The second measure was based on the rate of firing activities. At the beginning, the driven rate at each ISI was calculated by subtracting the mean of spontaneous firing rate from firing rate for 50–500 ms after the onset of the click trains. Then, the driven rate was filtered by a weighted average with its 5 neighbors in the ratio of 1:2:3:2:1 and was plotted against ISI (hereafter, "rate modulation transfer function [rMTF]"; Fig. 1A–3). When this measure exceeded the threshold for excitation (= 2*SD of the mean of spontaneous firing rate; dotted line), we evaluated rate response taking place (solid bar).

To estimate the recovery time course of neural responsivity, we presented a single click (= probe stimulus) at 1.0, 1.8 or 3.6 s after the termination of the click trains and measured the number of evoked spikes (#spikes) (Fig. 4D). For simplicity, we set ISI of the click trains at either best ISI or 0.9 multiples of the *γ*-*δ *border (see Results).

### Single-click paradigm

To assess the involvement of suppressory processes in the stimulus-locking responses, we conducted a single-click paradigm: a single rectangular click (1-ms duration, at the best SPL) was presented 10–20 times at intervals of 4.0–5.5 s. The spike occurrence was examined with dot raster plots (Fig. 6A–1) and post-latency time histogram (Fig. 6B–2). For constructing the latter, we first obtained peri-stimulus time histogram (bin width = 2 ms). We defined the response latency as the beginning of three consecutive bins in which the firing rate exceeded the threshold for excitation (see above). Then, we obtained a post-latency time histogram for 500 ms after the latency (bin width = 10 ms). The threshold for "suppression" was set at the mean minus 2*SD of the spontaneous firing rate (dotted line, bottom). Note that this paradigm was executed only for part of the AI neurons (*n *= 35) that showed an appreciable amount of spontaneous firing rate to make the threshold for suppression > 0 (spikes/s).

### Statistics

Physiological data was presented as the mean ± SD. If necessary, #spikes was normalized to the value elicited by the 1st click in the train that was delivered at the best ISI (= control level; e.g., Fig. 4C). In general, we employed Student's *t*-test or a one-way repeated Analysis of variance (ANOVA) followed by (post-hoc) Fisher protected least-significant difference test (PLSD) for pairwise comparisons. The significance level was set at *P *< 0.05 against a null hypothesis of equal performance.

### Minimal cortical models

We adopted a single-cell dynamic model for describing the response kinetics. Simulation of the model was performed using XPPAUT, developed by G.B. Ermentrout and available at . Although the model is minimal, it produces a good approximation of the spike shapes and temporal response properties experimentally observed in the present study. The principal equation describing the change in the membrane potential *V*_*m *_(mV) of a neuron at the soma is given by the following current balance equation:

(1) *C*_*m *_*dV*_*m*_/*dt = I*_*ion *_*+ I*_*syn *_*+ I*_*app *_*+ noise*

where *C*_*m *_is the membrane capacitance (1 *μ*F/cm^2^). The right-hand side incorporates the intrinsic ionic currents (*I*_*ion*_), synaptic currents (*I*_*syn*_) and external input (*I*_*app*_). In addition, the model includes noise current that comes from a presynaptic neuron (*noise*; *λ *= 500 Hz). The presynaptic neuron fires randomly with a uniform distribution in time. The usual method of integration was a fourth-order Runge-Kutta method with a time step of *dt *= 0.02.

#### Intrinsic Current

*I*_*ion *_(equation 1) represent the sum of intrinsic ionic currents which are contributed mainly by the voltage-dependent sodium current (*I*_*Na*_), potassium current (*I*_*K*_) and leak current (*I*_*leak*_).

(2) *I*_*ion *_= *I*_*Na *_+ *I*_*k *_+ *I*_*leak*_

Each current was modeled in terms of the Hodgkin and Huxley type first-order kinetic [62].

(3) $$\begin{array}{l} Sodiumcurrent(I_{Na})\\ \;\;\;\;\;\;\;\;\;\;\;\;\;\;\;\;\;\;\;\;\;\;\;\;\;\;\;\;\;\;\;\;\;\;\;\;\;I_{Na}={\bar{g}}_{Na}m^{\text{p}}h^{q}(V_{m}-E_{Na}) \end{array}$$

where the values of the parameters were derived from the experimental data and other models [63,64] (same as *I*_*k *_and *I*_*leak*_) and are as follows: $\bar{\text{g}}$_*Na *_(maximal conductance of the sodium channel) = 120 mS/cm^2^, *p *= 3, *q *= 1, *E*_*Na *_= 50 mV.

The equation for the *I*_*Na *_activation variable is:

(4) *dm*/*dt *= (*m*_∞ _- *m*)/*τ*_*m*_

where *m*_∞ _is the equilibrium value and *τ*_*m *_is a time constant of *m *as a function of *V*_*m*_,

(5) *m*_∞ _= *α*_*m*_(*V*_*m*_)/[*α*_*m*_(*V*_*m*_) + *β*_*m*_(*V*_*m*_)]

(6) *τ*_*m *_= [*α*_*m*_(*V*_*m*_) + *β*_*m*_(*V*_*m*_)]^-1^

with the forward and backward rate constants being given by:

(7) *α*_*m*_(*V*_*m*_) = 0.1 (*V*_*m *_*+ *40)/{1 - *exp *[- (*V*_*m *_+ 40)/10]}

(8) *β*_*m*_(*V*_*m*_) = 4 *exp *[- (*V*_*m *_+ 65)/18]

The equation for the *I*_*Na *_inactivation variable is:

(9) *dh/dt *= (*h*_∞ _- *h*)/*τ*_*h*_

where *h*_∞ _is the equilibrium value and *τ*_*h *_is a time constant of *h *as a function of *V*_*m*_,

(10) *h*_∞ _= *α*_*h*_(*V*_*m*_)/[*α*_*h*_(*V*_*m*_) + *β*_*h*_(*V*_*m*_)]

(11) *τ*_*h *_= [*α*_*h*_(*V*_*m*_) + *β*_*h*_(*V*_*m*_)]^-1^

with the forward and backward rate constants being given by:

(12) *α*_*h*_(*V*_*m*_) = 0.07 *exp *[- (*V*_*m *_+ 65)/20]

(13) *β*_*h*_(*V*_*m*_) = {*1 + exp *[- (*V*_*m *_*+ *35)/10]}^-1^

(14) $$\begin{array}{l} Potassiumcurrent(I_{K})\\ \;\;\;\;\;\;\;\;\;\;\;\;\;\;\;\;\;\;\;\;\;\;\;\;\;\;\;\;\;\;\;\;\;\;\;\;\;\;\;\;\;I_{k}={\bar{g}}_{k}n^{\text{j}}(V_{m}-E_{k}) \end{array}$$

where $\bar{\text{g}}$_*k *_= 36 mS/cm^2^, j = 4, *E*_*k *_= *-*77 mV.

The equation for the *I*_*K *_activation variable is:

(15) *dn*/*dt *= (*n*_∞ _- *n*)/*τ*_*n*_

where *n*_∞ _is the equilibrium value and *τ*_*n *_is a time constant of *n *as a function of *V*_*m*_

(16) *n*_∞ _= *α*_*n*_/(*α*_*n *_+ *β*_*n*_)

(17) *τ*_*n *_= (*α*_*n *_+ *β*_*n*_)^-1^

with the forward and backward rate constants being given by:

(18) *α*_*n*_(*V*_*m*_) = 0.01 (*V*_*m *_+ 55)/{1 - *exp *[- (*V*_*m *_+ 55)/10]}

(19) *β*_*n*_(*V*_*m*_) = 0.125 *exp *[- (*V*_*m *_+ 65)/80]

(20) $$\begin{array}{l} Leakcurrent(I_{Leak})\\ \;\;\;\;\;\;\;\;\;\;\;\;\;\;\;\;\;\;\;\;\;\;\;\;\;\;\;\;\;\;\;\;\;\;\;\;\;I_{leak}=g_{L}(V_{m}-E_{L}) \end{array}$$

where g_*L *_= 0.3 mS/cm^2 ^and *E*_*L *_= *-*54.4 mV.

#### Postsynaptic Current

*I*_*syn *_(equation 1) represents the sum of excitatory and inhibitory postsynaptic currents (EPSCs and IPSCs, respectively) which include the AMPA-mediated fast EPSCs (*I*_*AMPA*_), NMDA-mediated slow EPSCs (*I*_*NMDA*_), GABA_A_-mediated fast IPSCs (*I*_*GABAA*_), and GABA_B_-mediated slow IPSCs (*I*_*GABAB*_).

(21) *I*_*syn *_= *I*_*AMPA *_+ *I*_*NMDA *_+ *I*_*GABAA *_+ *I*_*GABAB*_

***AMPA-mediated current *(*I*_*AMPA*_) ***I*_*AMPA *_was modeled according to simple open/closed kinetics described by the following equation [65]:

(22) $$I_{AMPA}={\bar{\text{g}}}_{AMPA}s_{AMPA}(V_{m}-E_{glu\;})$$

here, the maximal conductance of the AMPA receptors, $\bar{\text{g}}$_*AMPA *_= 0.15 nS and the reversal potential of glutamate, *E*_*glu *_= 0 mV. A gating variable *s*_*AMPA*_, representing the fraction of the receptors in the open state, is modeled as:

(23) *ds*_*AMPA*_/*dt *= *σ*_*AMPA *_[*T*_*AMPA*_(*V*_*m*_)] (1 - *s*_*AMPA*_) - *s*_*AMPA*_/*τ*_*AMPA*_

We chose the value *σ*_*AMPA *_= 5 M^-1 ^s^-1^, *τ*_*AMPA *_= 2 ms which are within the range reported by Stern et al. (1992) [66]. The concentration of the released transmitter, [*T*_*AMPA*_(*V*_*m*_)], is a function of the membrane potential.

(24) [*T*_*AMPA*_(*V*_*m*_)] = [*T*_*AMPA*_]_*max*_/{1 + exp [- (*V*_*m *_- *θ*_*AMPA*_)/*K*_*AMPA*_]}

where [*T*_*AMPA*_]_*max *_= 1 mM, *θ*_*AMPA *_= -20 mV and *K*_*AMPA *_= 2 mV [67,68].

***NMDA-mediated current *(*I*_*NMDA*_) ***I*_*NMDA *_can be represented with a similar scheme as that for AMPA receptors, with a voltage-dependent magnesium block, *B*(*V*_*m*_).

(25) $$I_{NMDA}={\bar{\text{g}}}_{NMDA}s_{NMDA}(V_{m}-E_{glu})B(V_{m})$$

(26) *B*(*V*_*m*_) = {1 + exp(-0.062 *V*_*m*_) [Mg^2+^]_o_/3.57}^-1^

Here we chose $\bar{\text{g}}$_NMDA _= 0.38 nS and the external magnesium concentration, [Mg^2+^]_o _= 1 mM [69]. Since the rise time of *I*_*NMDA *_cannot be neglected, two differential equations are needed to model the kinetics. We used the equations proposed by Golomb et al. (2006) [70].

(27) *ds*_*NMDA*_/*dt *= *k*_*sn *_*x*_*NMDA *_(1 - *s*_*NMDA*_) - *s*_*NMDA*_/*τ*_*NMDA*_

(28) *dx*_*NMDA*_/*dt *= *k*_*xn *_[1 + tanh(*V*_*m*_/4)](1-*x*_*NMDA*_) - {1 - [1 + tanh(*V*_*m*_/4)]} *x*_*NMDA*_/~*τ*_*NMDA*_

where *k*_*sn *_= 1 ms^-1^, *τ*_*NMDA *_= 120 ms, *k*_*xn *_= 1 ms^-1 ^and ~ *τ*_*NMDA *_= 14 ms.

***GABA*_*A*_-*mediated current *(*I*_*GABAA*_) ***I*_*GABAA *_can be represented with a two-state model similar to that of the AMPA receptors. The postsynaptic current is given by

(29) $$I_{GABAA}={\bar{\text{g}}}_{GABAA}s_{GABAA}(V_{m}-E_{Cl})$$

where $\bar{\text{g}}$_*GABAA *_= 0.1 nS, *E*_*Cl *_= *-*80 mV. A gating variable *s*_*GABAA *_for a GABA_A _receptor, is modeled according to

(30) *ds*_*GABAA *_/*dt *= (1 - *s*_*GABAA*_) *σ*_*GABAA *_[1 + tanh(*V*_*pre*_/4)] - *s*_*GABAA *_/*τ*_*GABAA*_

*σ*_*GABAA *_= 2 and *τ*_*GABAA *_= 12 msec [71,72].

***GABA*_*B*_-*mediated current *(*I*_*GABAB*_) **The activation properties of GABA_B _receptors were based on the following steps: (1) the binding of GABA to the GABA_B _receptor, leading to the activated receptor; (2) the activated GABA_B _receptor catalyzes the activation of G-proteins on the intracellular side; and (3) the binding of activated G-proteins to open K^+ ^channels. These steps are described by the following equations [73]:

(31) $$I_{GABAB}={\bar{\text{g}}}_{GABAB}[s_{GABAB}^{\text{n}}/(s_{GABAB}^{\text{n}}+K_{d})](V_{m}-E_{k})$$

(32) *ds*_*GABAB*_/*dt *= *K*_1 _*r*_*GABAB *_- *K*_2 _*s*_*GABAB*_

(33) *dr*_*GABAB*_/*dt *= *K*_3 _[*T*_*GABAB*_] (1 - *r*_*GABAB*_) - *K*_4 _*r*_*GABAB*_

where $\bar{\text{g}}$_GABA_B__= 1.0 nS [74], *s*_*GABAB *_represents the normalized G-protein concentration in the activated form, *K*_*d *_refers to the dissociation constant of G-protein binding to K^+ ^channels, *r*_*GABAB *_is the fraction of the GABAB receptors in the activated form. GABA concentration in the synaptic cleft, [*T*_*GABAB*_], was set to 1 mM for 1 ms when the membrane potential crossed zero (only rising phase). Fitting this model to whole-cell recorded GABA_B _currents [65] gave the following values: *K*_*d *_= 100 *μ*M^4^, *K*_1 _= 180 s^-1^, *K*_2 _= 34 s^-1^, *K*_3 _= 9 × 10^4 ^M^-1^s^-1^, *K*_4 _= 1.2 s^-1 ^with *n *= 4 binding sites.

#### External Input

The external input (*I*_*app*_) was modeled as 1-ms-long repetitive square-wave pulses (initial amplitude = 12.5 *μ*Acm^-2^) with or without frequency-dependent depression which results mainly from temporal exhaustion of readily releasable neurotransmitter pool at presynaptic terminals [18].

Frequency-dependent depression was modeled according to the equation of Dobrunz and Stevens (1997) [75]:

(34) *P*(*N*) = 1 - exp(-*k N*^3/2^)

here *P *is the release probability and *N *is the number of readily releasable vesicles. We set *k *= 0.05 and the initial value of *N *= 5, then the above equation gives the initial release probability *P*_1 _= 0.43. *N *is modeled in terms of the depletion vs. refilling dynamics of the vesicles:

(35) *dN*/*dt *= -*λ*_*d *_*N *+ *λ*_*r *_(*N*_*c *_- *N*)

(36) *λ*_*d *_= *f*_*r*_/*n*_*d*_

where *λ*_*d *_and *λ*_*r *_are the depletion and refilling time constant, respectively, while *N*_*c *_represents the maximum size of the readily releasable vesicles, and *n*_*d *_represents the number of stimuli required to deplete the vesicles. Parameter values are set such that *λ*_*r *_= 0.05, *N*_*c *_= 15 and *n*_*d *_= 14. These values were chosen from the range given in previous reports [75,76] and were selected to fit to the present data that most probably reflects frequency-dependent depression (Fig. 4C, open circles). Since synaptic potency, which is defined as the average size of the synaptic response when transmitter release does occur, has been proven not to considerably vary despite the changes in release probability [75,77], we considered that *I*_*app *_is linearly related to *P*(*N*).

We have often observed, as already reported by Dobrunz and Stevens (1997) [75], that experimentally measured responses to the 2nd click far exceeded the calculated value from equation (34) especially at very short ISIs (i.e., "paired-pulse facilitation") possibly due to a residual elevation in the presynaptic intracellular calcium concentration [19,78]. To compensate this, we substituted the following equation to describe the release probability of the responses to the 2nd signal in the train, *P*_2_:

(37) *P*_2 _= *P*_1 _[*C*_1 _+ *C*_2 _exp(-*int*/*τ*_1_)]

here, *int *= 1000/*f*_*r*_. The best fit of this kinetic scheme to the present data (Fig. 5D, filled circle) gave *C*_1 _= 0.2,*C*_2 _= 1.3 and *τ*_1 _= 20 ms.

## Abbreviations

AI: primary auditory cortex; AMPA: alpha-amino-3-hydroxy-5-methyl-4-isoazolepropionic acid; ANOVA: analysis of variance; CV syllable: consonant-vowel syllable; EPSP: excitatory post-synaptic potential; E-S sequence: excitation-suppression sequence; $\bar{\text{g}}$: conductance; GABA: gamma-aminobutyric acid; IPSP: inhibitory post-synaptic potential; ISI: inter-stimulus intervals; MGB: medial geniculate body; NMDA:*N*-methyl-*D*-aspartate; PLSD test: protected least-significant difference test; rMTF: rate modulation transfer function; #spikes: the number of spikes; SPL: sound pressure level; tMTF: temporal modulation transfer function; VOT: voice onset time

## Competing interests

The authors declare that they have no competing interest.

## Authors' contributions

MS conceived and designed the study and wrote the paper. YS participated in the design of the study. MS performed most of the experiments. SC and LQ contributed methodology. All authors read and corrected the paper and added suggestions.
